# Serotonin biosynthesis as a predictive marker of serotonin pharmacodynamics and disease-induced dysregulation

**DOI:** 10.1038/srep30059

**Published:** 2016-07-21

**Authors:** Richard W. D. Welford, Magali Vercauteren, Annette Trébaul, Christophe Cattaneo, Doriane Eckert, Marco Garzotti, Patrick Sieber, Jérôme Segrestaa, Rolf Studer, Peter M. A. Groenen, Oliver Nayler

**Affiliations:** 1 Actelion Pharmaceuticals Ltd., Gewerbestrasse 16, CH-4123 Allschwil, Switzerland

## Abstract

The biogenic amine serotonin (5-HT) is a multi-faceted hormone that is synthesized from dietary tryptophan with the rate limiting step being catalyzed by the enzyme tryptophan hydroxylase (TPH). The therapeutic potential of peripheral 5-HT synthesis inhibitors has been demonstrated in a number of clinical and pre-clinical studies in diseases including carcinoid syndrome, lung fibrosis, ulcerative colitis and obesity. Due to the long half-life of 5-HT in blood and lung, changes in steady-state levels are slow to manifest themselves. Here, the administration of stable isotope labeled tryptophan (heavy “h-Trp”) and resultant *in vivo* conversion to h-5-HT is used to monitor 5-HT synthesis in rats. Dose responses for the blockade of h-5-HT appearance in blood with the TPH inhibitors L-*para*-chlorophenylalanine (30 and 100 mg/kg) and telotristat etiprate (6, 20 and 60 mg/kg), demonstrated that the method enables robust quantification of pharmacodynamic effects on a short time-scale, opening the possibility for rapid screening of TPH1 inhibitors *in vivo*. In the bleomycin-induced lung fibrosis rat model, the mechanism of lung 5-HT increase was investigated using a combination of synthesis and steady state 5-HT measurement. Elevated 5-HT synthesis measured in the injured lungs was an early predictor of disease induced increases in total 5-HT.

The biogenic amine serotonin (5-HT) is a biochemical messenger and regulator that signals through 13 receptors which are distributed throughout the nervous system and peripheral organs^1^,^2^,^3^. 5-HT was initially described as a vasoconstrictor and in the periphery it also plays a role in vasodilation, hemostasis, intestinal motility^4^, wound healing^5^ and inflammatory responses^6^. 5-HT is synthesized in two steps from dietary L-tryptophan (Trp) and accounts for 1–3% of Trp metabolism^7^. The first and rate limiting step of 5-HT production is hydroxylation catalyzed by the non-heme iron pterin-dependent oxygenase Tryptophan hydroxylase (TPH) (Fig. 1a). There are two TPH isoforms, TPH2 is mainly expressed in the central nervous system and enteric neurons, while TPH1 is expressed in the periphery and pineal gland^8^. The second step in 5-HT synthesis is rapid decarboxylation of 5-hydroxytryptophan (5-HTP) by the enzyme aromatic amino acid decarboxylase (DDC). Peripheral 5-HT is said to be largely synthesized by TPH1 expressed in the enterochromaffin cells lining the gut, where it is initially stored in secretory granules via the action of vesicular monoamine transporters^1^. 5-HT can then be released into the extracellular space and either signal through receptors or be taken-up in to other cell types that express the 5-HT re-uptake transporter SERT such as epithelial cells, smooth muscle cells and platelets. Platelet 5-HT has a half-life of at least 3 days and platelets are able to buffer plasma 5-HT complicating its measurement^9^,^10^. 5-HT is further metabolized intracellularly to 5-hydroxyindole acetic acid (5-HIAA) by a combination of the mitochondrial enzyme monoamine oxidase A (MAO-A) and an aldehyde dehydrogenase (AD). 5-HIAA is excreted in the urine and can be monitored as a surrogate of 5-HT level, with 24 hour urinary 5-HIAA used as a diagnostic for carcinoid syndrome^11^.

Increasing evidence implicates TPH1 in a number of peripheral diseases including lung fibrosis^12^,^13^, ulcerative colitis^14^, pulmonary hypertension^15^, osteoporosis^16^, irritable bowel syndrome^17^ and obesity^18^,^19^. Recently, the non-brain penetrating TPH inhibitor telotristat etiprate (LX-1032) has demonstrated clinical benefit in patients with carcinoid syndrome^20^,^21^.

Tracers, in particular stable isotope tracers, enable the collection of detailed kinetic data on flux through molecular systems *in vivo*, which can then be described in mathematical models^22^,^23^. Recent studies with stable isotope tracers have enriched our molecular understanding in the fields of Alzheimer’s disease and fibrosis by studying the kinetics of amyloid-beta and collagen respectively^24^,^25^,^26^,^27^,^28^. Previous *in vivo* studies investigating 5-HT synthesis employed tracers with some inherent disadvantages (*vide infra*), which we sought to overcome by using a ^13^C,^15^N stable isotope labeled Trp tracer ((^13^C_11_)Trp or (^13^C_11_,^15^N_2_)Trp) from now on collectively referred to as heavy-Trp (h-Trp)), accompanied by measurement with LC-MS/MS (Fig. 1b). The h-Trp used is metabolically nearly identical to normal Trp and can be considered as a quasi “label free” approach^29^,^30^. Additionally, no radioactivity is used, the analytics are highly specific, and simultaneous monitoring of both naturally occurring and labeled compound results in significantly improved data quality and richer information^29^. In comparison, chemically modified Trp analogues, such as methyl^31^,^32^ and fluoro^33^,^34^ Trp suffer from altered metabolism compared to the parent. Radioactive tracers complicate *in vivo* work, analytics and analysis, with selectivity challenges arising due to the complexity of Trp metabolism and the requirements for a second method to measure unlabeled molecule^35^. In published studies using deuterated-Trp as a tracer the label was lost by back exchange^36^ and insights into 5-HT biology were limited as only the 5-HIAA metabolite could be quantified due to background interference^37^. During the process of completing the work described here, a single study using (^15^N_2_)Trp in rats with monitoring of labeled 5-HT by chemical derivatization and GCMS was published, although only a single biological condition was tested^38^.

Here, h-Trp was administered to rats and the conversion to h-5-HT was monitored to measure 5-HT synthesis. Pharmacodynamics and disease effects on 5-HT synthesis could be observed long before steady state 5-HT levels were altered. Monitoring of 5-HT synthesis was demonstrated to enable medium through-put testing of TPH1 inhibitors *in vivo* and was used to explore the mechanism of 5-HT dysregulation in a bleomycin-induced model of lung fibrosis.

## Materials and Methods

### Chemicals

The tracers (^13^C_11_)Trp and (^13^C_11_,^15^N_2_)Trp were from Cambridge Isotope Laboratories (Andover, USA) and Campro Scientific (Germany) respectively. The internal standards (^2^H_5_)Trp and (^2^H_4_)5-HT were from C/D/N Isotopes (Canada) and (^2^H_5_)5-HIAA from EQ Laboratories (Germany). Standards of (^13^C_10_)5-HT and (^13^C_10_,^15^N_2_)5-HT were synthesized on a small scale from their respective labeled Trp using a combination of DDC (RnD Systems, UK, prod no 3564-DC) and in-house purified TPH1^39^. The concentration of the labeled 5-HT standard was determined using HPLC with a fluorescence detector with 5-HT as a reference for the standard curve. LX-1032 (telotristat etiprate) was synthesized at Synphabase (Switzerland). All other chemicals were from Sigma-Aldrich.

### Animal work

All animal studies were conducted in accordance with Swiss Animal Protection Laws, conform to Directive 2010/63/EU of the European Parliament on the protection of animals under scientific purposes, and was specifically approved by Basel-Landschaft Cantonal Veterinary Office under license 169 and 371. Male Wistar rats (190–275 g) were purchased from Harlan Laboratories B.V. (Venray, Netherlands). All animals were housed in climate-controlled conditions with 12-hour light/dark, maintained under identical conditions and had free access to normal pelleted rat chow and drinking water.

Oral h-Trp studies were performed with oral gavage of either (^13^C_11_)Trp or (^13^C_11_,^15^N_2_)Trp in an 0.5% methyl cellulose, 0.5% Tween-80 solution at a dose of 6 mg/mL (volume of administration 5 mL/kg). Administration of h-Trp is defined as time = 0. The TPH inhibitors, (L-*para*-chlorophenylalanine (PCPA) or LX-1032) or vehicle (0.5% methyl cellulose, 0.5% Tween-80 solution) were administered at t = −1 hour before h-Trp gavage. Reported doses for LX-1032 are those of the active agent (counter ion not included). At the time points indicated in the figures, the rats were sampled for blood via a sublingual puncture under isofluran-induced narcosis (3%). EDTA-blood samples were either frozen directly for measurement of 5-HT and metabolites or converted to plasma for measurement of TPH inhibitor concentrations. To perform the h-Trp infusion study (concentration at steady state of 500 ng/mL), a saline stock solution of h-Trp (5 mg/mL) was injected as an *intravenous* bolus (1.1 mg/kg in 30 seconds), followed by a constant rate of infusion of 0.75 mg/kg.hour (volume of injection 1 mL/kg). Over a 10-hour time period a total dose of 7.5 mg/kg of h-Trp was injected. In the infusion study LX-1032 or vehicle was administered by gavage 30 minutes prior to the start of the infusion (defined as t = 0). In the disease context of pulmonary fibrosis, saline or bleomycin solutions were instilled using an intra-tracheal micro-sprayer (Model IA-1B-R, Penn-Century Inc., Wyndmoor, USA). Control animals received 1 mL/kg of sterile saline followed by 1 mL/kg of air. Bleomycin-treated rats received a single dose of sterile bleomycin sulphate (1.5 mg/kg) dissolved in 1 mL/kg of saline, also followed by 1 mL/kg air to distribute the drug equally throughout the lungs. At the dedicated time points, rats were anesthetized (isofluran 5%) and euthanized by exsanguination at 7, 14 , 21 and 28 days after the instillation. After the terminal blood collection, the lungs were removed and snap frozen prior to lung hydroxyproline measurements (right middle lobe), 5-HT content assessment (blood and accessory lobe) and gene expression evaluation (right cranial lobe).

### Bioanalytical sample preparation for 5-HT pathway metabolites

Organ samples were homogenized using a turrax with a 1/6 (w/v) dilution in 0.5 M acetic acid. Homogenates were cleared by centrifugation and the supernatant stored at −80 °C prior to analysis. Ten point calibration curves containing (concentration of highest calibrant) Trp (100 μM), h-Trp (20 μM), 5-HT (5 μM), h-5-HT (0.4 μM) and 5-HIAA (0.4 μM) were made up in 50 mg/mL BSA in PBS, with the highest concentration serial 2-fold diluted. Blood, calibrant and quality control samples (20 μL), were diluted by adding 140 μL water containing the internal standards ((^2^H_5_)Trp 50 nM; (^2^H_4_)5-HT 10 nM; (^2^H_5_)5-HIAA 10 nM). Organ homogenates were similarly diluted. Following mixing, 480 μL acetonitrile was added to precipitate proteins. Samples were again mixed, cleared by centrifugation, 460 μL supernatant transferred to a new plate, dried under a stream of heated N_2_ and reconstituted in 230 μL water for LC-MS/MS analysis. For blood experiments 10 μL was injected into the LC-MS/MS corresponding to 0.625 μL of blood.

### LC-MS/MS analysis for 5-HT pathway metabolites

LC-MS/MS analysis was performed using a Dionex UltiMate^®^ 3000 HPLC system with a QTRAP^®^5500 mass spectrometer (ABSciex™). A Luna^®^ C18(2), 3 μm, 100 Å, 100 × 2 mm (Phenomenex^®^) column was used at a flow rate of 0.3 mL/min at a temperature of 35 °C with buffer A: 5 mM ammonium formate containing 0.01% trifluoroacetic acid and buffer B: methanol. A 2 minute gradient from 5% to 55% buffer B was followed by a second 2 minute gradient from 55% to 80% buffer B. Finally the column was cleaned and re-equilibrated. The QTRAP®5500 was run in positive ion electrospray with the following settings: curtain gas (20), CAD (medium), source temperature (550), gas 1 (50), gas 2 (50), ion source voltage (5500), EP (10) and CXP (10). The following MRM transitions (Q1/Q3) were used for quantitation ((^13^C_11_,^15^N_2_)Trp tracer) 5-HT (178/161), h-5-HT (171/142.1), Trp (206/147), h-Trp (218/156), 5-HIAA (192/146) and h-5-HIAA (203/156). DP and CE were optimized for individual compounds using standard procedures. In some cases a second qualifier MRM transition was also monitored.

### Data analysis

**Figure 1:**
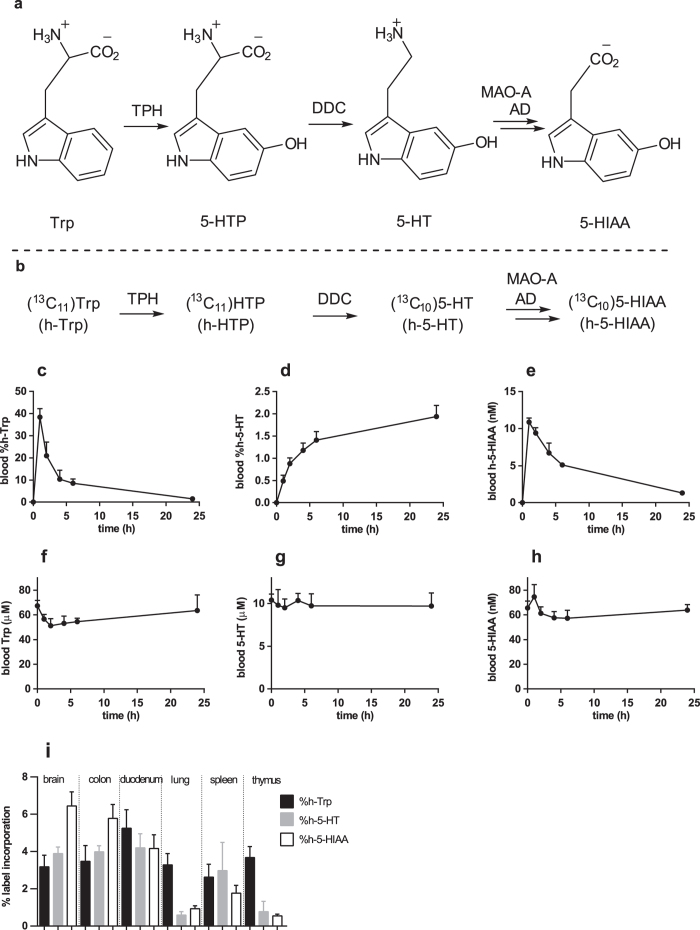
Biosynthesis and metabolism of 5-HT and h-5-HT. **(a, b)** Scheme for biosynthesis and metabolism of 5-HT and h-5-HT. **(c-h)** Kinetics of Trp, 5-HT, 5-HIAA and their stable isotope labeled counterparts in rats after administration of 30 mg/kg h-Trp at t = 0 h (n = 4, ±SD). **(c)** Blood %h-Trp; **(d)** blood %h-5-HT; **(e)** blood h-5-HIAA; **(f)** blood Trp; **(g)** blood 5-HT; **(h)** blood 5-HIAA. **(i)** From a separate experiment, the % of heavy labeled analyte in different organs 10 hours after oral administration of h-Trp (n = 5, ±SD).

The MRM chromatograms were processed using MultiQuant™ software (ABSciex™). Internal standard normalized calibration curves were fit to the simplest model possible and used to calculate concentrations. h-5-HIAA was quantified using a surrogate analyte approach with the 5-HIAA calibration curve. Additional calculations and statistics were performed using Microsoft Excel 2010 or GraphPad Prism 6 for graphed data and h-Trp tracer pharmacokinetics. When there is little change in unlabeled analyte, the data on stable isotope labeled analytes is presented as mole percent excess e.g. %h-Trp = 100^*^[h-Trp]/([h-Trp] + [Trp]). If similar changes in both labeled and unlabeled analyte occur mole percent excess is not used to avoid normalizing away relevant changes. Unless otherwise stated data is reported as average ± standard deviation. Oral h-Trp pharmacokinetic parameters were derived using the vehicle group (n = 4) from Fig. 1 and the vehicle group from 4 additional repetitions of this study. The fractional synthesis rate of h-5-HT given in the text was calculated from the vehicle group of the h-Trp infusion experiment by fitting the %h-5-HT points between 2 and 10 h for each rat with a linear regression and dividing by the average %h-Trp for that rat in the same period and are given in h^−1 ^40. Sample sizes for individual studies are given in the legend for each figure.

### Lung collagen content evaluation

Right middle lung lobe was reduced to small pieces with a scalpel blade. After homogenization in 2 mL of pure water with UltraTurrax^®^, Trichloracetic acid 50% (Fluka 91228) was added for proteins precipitation. After centrifugation (10 min at 4000 g), the pellet was hydrolyzed for 4 hours at 130 °C in 6N HCl followed with a neutralization step with NaOH 2.5 M pH 6–8 and filtration with a 0.45 μm filter unit (Millex^®^). Hydroxyproline (HYP) concentrations in the acid hydrosylates were measured using LC-MS/MS. Two-fold serial diluted calibrants from 2000 μg/mL down to 12.5 μg/mL were made up in 50-fold diluted rat lung acid hydrosylate. Then 20 μL sample was added to 580 μL of water, samples were mixed, then 20-fold further diluted with acetonitrile, again mixed, briefly centrifuged and transferred for injection on to the LC-MS/MS system. For each sample and calibrant 5 μL was injected on to an Acquity UPLC Amide 100 × 2.1 mm, 1.7 μm (Waters, Part Number: 186004801) column running at 0.5 mL/min with buffer A (water/acetonitrile 60/40 containing 100 mM ammonium formate, pH 4.5) and buffer B (water/acetonitrile 10/90 containing 10 mM ammonium formate pH 4.5). The column was run isocratically at 95% buffer B for 0.5 mins before a gradient to 50% buffer B over 4 minutes. Quantification was performed using a QTRAP^®^6500 mass spectrometer (ABSciex™) in positive ion electrospray with the following settings: curtain gas (25), CAD (high), source temperature (550), gas 1 (55), gas 2 (50), ion source voltage (5500), EP (10) and CXP (10) with the following MRM transitions for HYP (Q1/Q3) (132/68).

Methodology for qPCR, inhibitor quantification and the BON cell assay are given in the supplementary information provided with the online version of this manuscript.

## Results

### Tracer selection and method specificity

A number of factors need to be taken into account when selecting a suitable h-Trp tracer for *in vivo* studies. Deuterium containing Trp tracers were avoided as they have the potential to back exchange leading to label loss^36^ and to generate large primary kinetic isotope effects (classically up to 7), leading to perturbation of the system by the tracer. Therefore tracers containing ^13^C and ^15^N isotopes, which will have negligible kinetic isotope effects (<1.1), were utilized. For 5-HT, the M + 1 and M + 2 ions have natural abundances of 11% and 1.1% respectively, resulting in a significant background signal if the h-Trp administered contains one or two labels. Based on the additional criteria of ready commercial availability and cost, the studies described used either (^13^C_11_)Trp or (^13^C_11_^15^N_2_)Trp as a tracer, collectively referred to as h-Trp. Trp, 5-HT and 5-HIAA along with their stable isotope labeled counterparts were analyzed using liquid chromatography tandem mass spectrometry. Prior to h-Trp administration, biosamples from rats showed clean, blank chromatograms for h-Trp, h-5-HT and h-5-HIAA, indicating the absolute analytical specificity of the approach (Supplementary Figure 1).

### h-Trp and h-5-HT blood pharmacokinetics and h-5-HT production in organs

Initially the conversion of h-Trp to h-5-HT was studied in rats. Based on the standard Trp consumption of 400 mg/kg/day, the estimated half-life of 5-HT and assay sensitivity, an oral dose of 30 mg/kg h-Trp was chosen to assess the pharmacokinetics of h-Trp and conversion to h-5-HT and h-5-HIAA. h-Trp appeared rapidly in blood and peaked with a C_max_ = 32 ± 6.6 μM and t_max_ = 1 h (AUC = 130 ± 13 μM*h) (values derived from 5 independent repeated measurements each with n = 4) (Fig. 1c). h-5-HT increased rapidly and linearly during the first 4 hours after h-Trp administration (Fig. 1d). After 6 hours, concomitant with a drop of h-Trp levels, the h-5-HT synthesis rate decreased and h-5-HT levels reached a plateau. Newly synthesized h-5-HT was cleared from blood with a t_1/2_ of 2.5 ± 0.3 day (Supplementary Figure 2). The h-5-HT metabolite h-5-HIAA reached its maximal concentration at 1 hour and was subsequently cleared over the following 24 hours (Fig. 1e). During the same observation period, the total blood concentrations of unlabeled 5-HT remained constant, while Trp and 5-HIAA have minimal diurnal variation (Fig. 1f–h). Using a constant infusion of h-Trp in rats a fractional synthesis rate of 0.021 ± 0.001 h^−1^ for h-5-HT was calculated (n = 6, ±SD). The levels of newly synthesized h-Trp, h-5-HT and h-5-HIAA in tissues were assessed in rats that were sacrificed 10 hours after oral h-Trp administration. In the gut and the brain the mole percent labeled Trp (%h-Trp), %h-5-HT and %h-5-HIAA were quite similar (Fig. 1i). In the lung and the thymus differences were more pronounced, with %h-5-HT ≈ %h-5-HIAA ≪ %h-Trp, indicating minimal basal 5-HT synthesis in these organs.

### Pharmacological inhibition of h-5-HT synthesis

**Figure 2:**
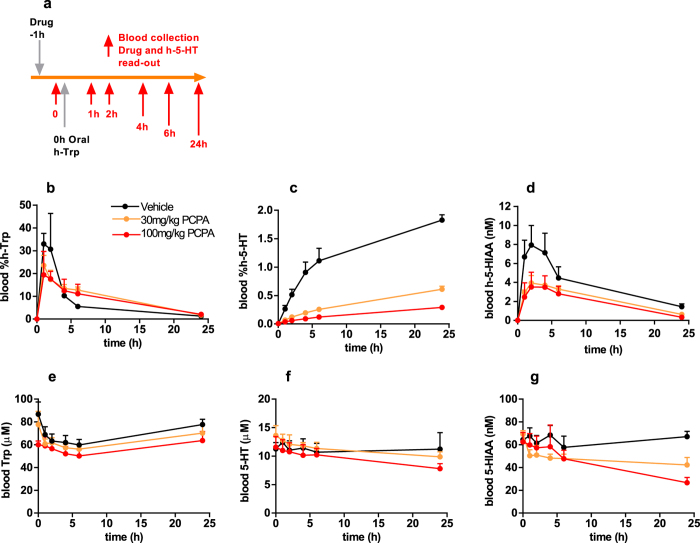
Inhibition of h-5-HT and h-5-HIAA appearance in rat blood by PCPA after oral h-Trp administration. **(a)** Experimental design of oral h-Trp study. Effect of 30 and 100 mg/kg PCPA on analytes in blood with time **(b)** %h-Trp; **(c)** %h-5-HT; **(d)** h-5HIAA; **(e)** Trp; **(f)** 5-HT; **(g)** 5-HIAA (n = 4, ±SD).

**Figure 3:**
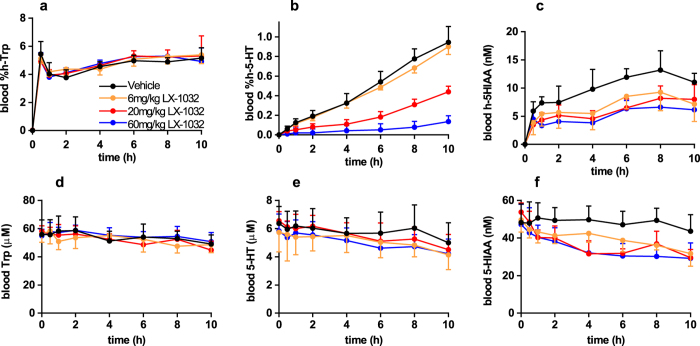
Inhibition of h-5-HT and h-5-HIAA appearance in rat blood by LX-1032 during h-Trp infusion. LX-1032 was dosed 30 min before a bolus of h-Trp at t = 0, followed by h-Trp infusion to maintain a constant h-Trp level during the experiment. Effect of 6, 20 and 60 mg/kg LX-1032 on analytes in blood with time **(a)** %h-Trp; **(b)** %h-5-HT; **(c)** h-5HIAA; **(d)** Trp; **(e)** 5-HT; **(f)** 5-HIAA (n = 6, ±SD).

The kinetic profile of h-5-HT appearance in the blood after oral h-Trp administration is reminiscent of an *in vitro* enzyme assay and suggested that the *in vivo* conversion of h-Trp to h-5-HT could be used to quantify pharmacodynamic effects of TPH inhibitors on a short timescale. Two known prototypic TPH inhibitors, PCPA^41^ and LX-1032^14^, which has an active metabolite *in vivo*, were selected and their inhibitory potency was confirmed using an *in vitro* whole cell assay (Supplementary Table 1). In rats, the inhibitors were dosed 1 hour prior to oral administration of h-Trp such that drug concentrations could be measured at the time of h-Trp administration (Fig. 2a). Both compounds inhibited the appearance of blood h-5-HT, with large measurable effects already one hour after dosing (Fig. 2c and Supplementary Figure 3b), while 5-HT was relatively unaffected even after 24 h (Fig. 2f and Supplementary Figure 3e). Both PCPA and LX-1032 also reduced blood h-5-HIAA (Fig. 2d and Supplementary Figure 3c), but the magnitude of the effect was smaller than for h-5-HT. In a follow-up dose response experiment a combination of h-Trp bolus and subsequent infusion was used to extend the time of linear h-5-HT production. LX-1032 was orally administered 30 minutes prior to commencing h-Trp administration and dose-dependent inhibition of h-5-HT was observed (Fig. 3a,b). Notably, the extent of inhibition by LX-1032 was independent of the h-Trp administration method for early time points. The plasma exposure of PCPA was far greater than that of the more potent LX-1032, although the known active metabolite of the latter was more highly exposed than its parent compound (Supplementary Table 1).

### Use of h-5-HT production to investigate the mechanism of 5-HT increase in a bleomycin-induced lung fibrosis model

**Figure 4:**
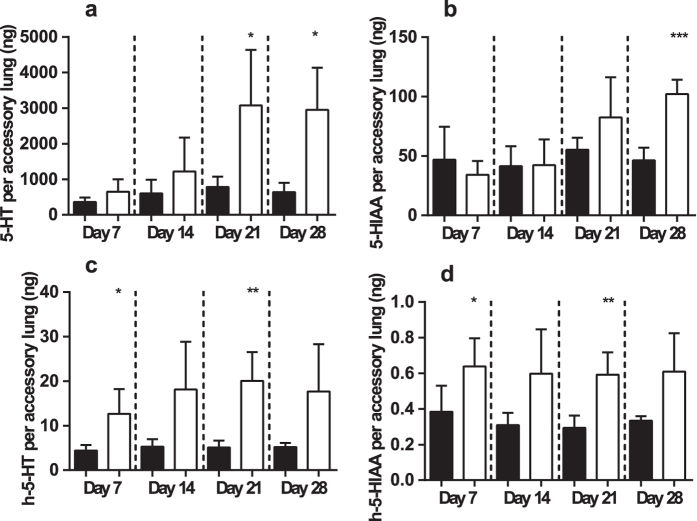
Accessory lung 5-HT, 5-HIAA h-5-HT and h-5-HIAA at 7, 14 ,21 and 28 days after disease induction in the bleomycin induced model of lung fibrosis in Wistar rats **(a–d)**. Black and white bars are NaCl and bleomycin treated animals respectively (n = 4–6 ± SD). A two tailed student’s t-test comparing bleomycin to NaCl for individual time points is given; *p < 0.05, **p < 0.01, ***p < 0.001.

To investigate the potential of h-5-HT to predict disease-induced changes in 5-HT a time-course study of the bleomycin-induced rat lung fibrosis model was performed^42^. Oral h-Trp was administered 4 hours prior to sacrifice in bleomycin- and saline-treated control animals and at each time point 5-HT, Trp, 5-HIAA, h-Trp and *de novo* synthesized h-5-HT and h-5-HIAA were quantified in lung tissue. Changes in body weight, lung weight and collagen confirmed induction of disease, with significant collagen increases being observed from day 14 (Supplementary Figure 4). In the bleomycin-treated group, lung 5-HT was only significantly elevated at day 21 (p = 0.029) and day 28 (p = 0.027) (Fig. 4a). In contrast, a statistically significant increase of newly synthesized lung h-5-HT was already observed at day 7 (p = 0.013) and remained elevated at day 14, 21 and 28 in bleomycin-treated animals (Fig. 4c). A similar effect was noted when comparing h-5-HIAA to 5-HIAA (Fig. 4b,d).

To probe the mechanism of 5-HT increase in the model, the lung expression of 69 genes involved in fibrotic processes and the 5-HT pathway was measured. The *Tph1* gene was upregulated by bleomycin administration, while *Slc6a4 (Sert*) was down regulated (Supplementary Figure 5). A Spearman correlation of each metabolite read-out was performed against each of the 69 genes for the bleomycin treated animals (n = 20) and the same analysis was performed for the NaCl treated animals for genes of interest. Statistically significant gene expression to metabolite level correlations were found for 5-HT, h-5-HT and 5-HIAA but not for their precursor Trp, although it was also increased by bleomycin (Table 1). The most significant correlations were between 5-HT, h-5-HT and 5-HIAA and genes with known critical roles in controlling 5-HT levels (Table 2). The lung level of 5-HT strongly correlated with both *Tph1* and *Slc6a4* mRNA level in the bleomycin treated animals (Table 2). New 5-HT in the lung (h-5-HT) was also most strongly correlated with the *Tph1* gene. In the saline treated animals *Slc6a4* did not significantly correlate with lung 5-HT (p = 0.57) or 5-HIAA (p = 0.96). However, when bleomycin was administered the levels of *Slc6a4* mRNA were significantly reduced, with the magnitude of reduction diminishing over the duration of the study (Supplemental figure 5b) resulting in a strong positive correlation between *Slc6a4* and both lung 5-HT and lung 5-HIAA (Table 2).

## Discussion

The administration of h-Trp enables and simplifies the tracking of *de novo* synthesis of 5-HT and its metabolite 5-HIAA *in vivo*. Here we show that *de novo* synthesized h-5-HT can be used as a predictive biomarker for 5-HT formation with only negligible perturbation to the system studied. The methodology is of particular utility in following accumulation of 5-HT in the blood, as this process has a t_1/2_ ~2.5 days in rats and longer in larger mammals^10^. The ability to track changes of h-5-HT in accessible biofluids with time, combined with the opportunities provided by utilizing more than one isotope of h-Trp vastly expands the options for studying the kinetics of 5-HT production, inhibition via TPH inhibitors, and regulation in disease.

The data confirms that Trp conversion to 5-HT in brain and gut is rapid, in good agreement with the known expression profiles of TPH1 and TPH2. In the lung the 5-HT levels were similar to those in the gut, but much lower label incorporation from h-Trp into h-5-HT or h-5-HIAA was observed, demonstrating that lung 5-HT turnover is very slow in a healthy rats. Low turnover of lung 5-HT under normal conditions is supported by high SERT expression^43^ and active up-take of 5-HT by endothelial and pulmonary smooth muscle cells^44^,^45^.

The amino acid PCPA is a non-specific inhibitor of TPH^46^ that crosses the blood brain barrier^47^. PCPA has been shown to lower urinary 5-HT and 5-HIAA in a carcinoid syndrome patient^48^ and in healthy rats it can reduce blood, gut and brain 5-HT^41^,^47^. The data presented here demonstrate that PCPA is a highly effective inhibitor of 5-HT synthesis in rats. LX-1032 and its systemically available metabolite^20^ have been observed to lower blood 5-HT by around 50% in mice^14^. A dose-dependent inhibition of h-5-HT biosynthesis upon single oral administration of PCPA or LX-1032 was observed supporting the monitoring of blood h-5-HT after h-Trp administration as an accurate predictor of whether compounds will be able to lower blood 5-HT. The magnitude and statistical significance in reductions of blood %h-5-HT were vastly superior to those for the 5-HT metabolite 5-HIAA, indicating the clear benefits of the stable isotope tracer approach. Using the oral administration of h-Trp it is possible to screen new pharmacological agents *in vivo* for inhibition of the appearance of blood h-5-HT with a reasonably high-through-put. Additionally, using the h-Trp tracer it is no longer necessary to sacrifice animals and examine gut to see a full effect on 5-HT levels as was performed in other studies^49^,^50^.

5-HT is known to be pro-fibrotic in situations of chronic injury by diverse mechanisms including TGF-beta activation^5^,^51^. Previously, lung 5-HT was shown to be increased in a mouse model of lung fibrosis^12^ and HTR2A/B receptor antagonists have shown anti-fibrotic effects in the same model^12^,^13^. In the bleomycin-lung fibrosis rat model, total lung 5-HT and new 5-HT synthesis (h-5-HT) were significantly correlated with lung *Tph1* mRNA, strongly suggestive of a contribution from local synthesis. The relevance of these correlations is strongly supported by their high ranking amongst the measured genes. Local 5-HT synthesis is likely further enhanced by the increase in lung Trp. The strong down regulation of *Slc6a4 (Sert*) upon bleomycin administration and its positive correlations with lung 5-HT and 5-HIAA suggests that local concentrations of both metabolites also become at least partially limited by *Slc6a4*. The reduction in *Slc6a4* is likely to increase extracellular 5-HT in the lung, increasing its availability for signaling through HTR2B, HTR2A, with the lung unable to maintain homeostatic 5-HT metabolism. Additionally, high levels of extracellular 5-HT are known to reduce surface expression of SERT on platelets, albeit through alteration in the trafficking dynamics of the protein^9^,^52^. The increases in lung h-5-HT and h-5-HIAA are visible at earlier time-points during progression of the disease model than their naturally occurring counterparts, indicating the ability of the stable isotope tracer technology to be a sensitive early predictor for the observation of disease induced regulations.

Stable isotope tracers of endogenous molecules have been extensively utilised in clinical studies for the measurement of rates due to their safety, high analytical specificity and the benefits of measuring both tracer and tracee^29^,^30^,^53^. Previously, using L-[*ring*-^2^H_5_]Phe it was shown that patients with phenylketonuria have reduced Phe to Tyr hydroxylation a reaction similar to that catalyzed by TPH^54^. In the future we plan to assess clinical utility of the presented h-Trp methodology. Given the important role of 5-HT in the central nervous system, it may be of interest to use h-Trp to study 5-HT synthesis in the brain. However, the necessity for invasive lumbar puncture could limit clinical utility compared to existing PET tracers, albeit with their own disadvantages^55^.

The benefits of using a stable isotope tracer approach to study peripheral *de novo* 5-HT synthesis and its inhibition *in vivo* are demonstrated. We show that quantification of h-5-HT and h-5-HIAA in blood and tissues is simple, precise, highly specific and predictive of steady state changes in 5-HT. The method considerably reduced the time and amount of compound needed to observe inhibition of 5-HT production by small molecular weight compounds *in vivo*, facilitating the discovery of improved pharmacological agents. In the bleomycin-lung fibrosis rat model, early increases in lung h-5-HT and h-5-HIAA were found to be predictive for increases in their unlabeled counterparts at later time points. The presented methodology improves and simplifies the study of 5-HT production from a kinetic, regulation and inhibition perspective and similar studies could be performed in man to improve our translational understanding.

## Additional Information

**How to cite this article**: Welford, R. W. D. *et al*. Serotonin biosynthesis as a predictive marker of serotonin pharmacodynamics and disease-induced dysregulation. *Sci. Rep.* **6**, 30059; doi: 10.1038/srep30059 (2016).

## Supplementary Material

Supplementary Information

## Figures and Tables

**Table 1:** Significant correlations between metabolites and genes in the lungs of bleomycin treated rats (n = 20).

**Metabolite**	**% Significantly positively correlated genes**	**% Significantly negatively correlated genes**
lung 5-HT (ng/lung)	27	17
lung h-5-HT (ng/lung)	3	1
lung 5-HIAA (ng/lung)	26	29
lung Trp (ng/lung)	0	0

A Spearmen correlation was performed for the listed metabolites against a panel of 69 genes. Number of significantly correlated genes per analyte. Genes with a p value < 0.05 were considered significantly correlated.

**Table 2:** 5-HT, h-5-HT and 5-HIAA levels are correlated with 5-HT related genes in the lungs of bleomycin treated rats (n = 20).

**Metabolite**	**Gene**	**Rank**	**Spearman r**	**p value**
lung 5-HT (ng/lung)	*Tph1*	1/69	0.7654	<0.0001
lung 5-HT (ng/lung)	*Slc6a4*	2/69	0.6932	0.0007
lung 5-HIAA (ng/lung)	*Htr2a*	1/69	−0.797	<0.0001
lung 5-HIAA (ng/lung)	*Slc6a4*	2/69	0.788	<0.0001
lung h-5-HT (ng/lung)	*Tph1*	1/69	0.5534	0.0114

A Spearmen correlation was performed for the listed metabolites against a panel of 69 genes and the r and p values for key top ranking correlations are given. Rank for the 69 genes was based on Spearman r (1 highest r, to 69 lowest r), after multiplication of negative correlations by −1.
