# Supplementary material for: Serotonin biosynthesis as a predictive marker of serotonin pharmacodynamics and disease-induced dysregulation
Source: Sci Rep. 2016 Jul 21;6:30059. doi: 10.1038/srep30059 (PMC4956766; doi:10.1038/srep30059)
Supplement: Supplementary Information [file srep30059-s1.pdf]

Supplemental Information

Serotonin biosynthesis as a predictive marker of serotonin pharmacodynamics and disease-induced dysregulation

Richard W. D. Welford*[a], Magali Vercauteren[a], Annette Trébaul, Christophe Cattaneo, Doriane Eckert, Marco Garzotti, Patrick Sieber, Jérôme Segrestaa, Rolf Studer, Peter M. A. Groenen and Oliver Nayler

Actelion Pharmaceuticals Ltd., Gewerbestrasse 16, CH-4123 Allschwil, Switzerland

a, These authors contributed equally to this work.

*Corresponding author: richard.welford@actelion.com

Supplementary methods

qPCR analysis of gene expression in the bleomycin model

The 69 gene panel was made up of 9 genes related to 5-HT, 26 genes for extracellular matrix synthesis and degradation, 12 genes from an idiopathic pulmonary fibrosis translational panel[1] and 22 genes from other fibrotic processes. Total RNA from the lung of the rats was isolated using the RNeasy mini kit according to the manufacturer's protocol (QIAGEN, Germany). Remaining genomic DNA was digested using the DNAse free kit (Ambion, USA). The quantity of RNA was analyzed using a Nanodrop spectrophotometer (Thermo Fisher, USA) and RNA quality was assessed using a Bioanalyzer 2100 (Agilent, USA). 1000 ng total RNA was reverse transcribed with the high capacity cDNA archive kit according to the manufacturer's protocol (Applied Biosystems, USA). The cDNA was preamplified according to the preamp kit with 14 cycles (Applied Biosystems, USA). QPCR was performed on an Biomark HD (Fluidigm, USA) using 96*96 dynamic arrays using Taqman assays **(Supplementary Table 2)** (Applied Biosystems, USA). Based on GENORM [2] 28s-Mm03682676_s1, Hprt1-Rn01527840_m1 and Pgk1-Rn00821429_g1 were used as reference genes. Expression values were calculated using a modified delta Ct method where an expression value of 1 reflects no detectable expression.

Quantitation of TPH inhibitors in plasma

PCPA, LX-1032 and LX-1032 metabolite were quantified by LC-MS/MS. Standard curves were generated in blank plasma (0.51-10,000 ng/mL). Samples were analyzed by a narrow-bore liquid chromatography method. A Luna® C8, 5 μm, 20 x 2.0 mm (Phenomenex®) column was used at a flow rate of 0.6 mL/min with mobile phase A: 0.1 % aqueous formic acid and mobile phase B: acetonitrile. A 0.15 minute isocratic step of 5% mobile phase B was followed by a 0.45 minute (0.25 minute for PCPA) gradient from 5% to 95% mobile phase B. Finally the column was cleaned and re-equilibrated. An API-4000 (ABSciex™) and an API 5000 (ABSciex™) Triple Quadrupole LC-MS/MS Mass Spectrometer, both equipped with a Turbo spray, were used for PCPA and LX-1032 (LX-1032 metabolite), respectively.

For PCPA, The plasma samples from the 30 mg/kg dose and the 100 mg/kg dose were diluted with blank plasma 30 and 100 times, respectively. Calibrant and samples were protein precipitated by addition of 3 volumes of methanol containing phenylalanine as an internal standard, cleared by centrifugation and diluted (1:1, v/v) with mobile phase. A 15 μL volume was injected on the LC-MS/MS.

For LX-1032 and LX-1032 metabolite, calibrant and plasma samples were stabilized with 0.1% dichlorvos (v/v) and diluted 3 times with blank plasma. Calibrant and samples were protein precipitated by addition of 3 volumes of methanol containing LX1031 as an internal standard, cleared by centrifugation and diluted (1:1, v/v) with mobile phase. A 3 μL volume was injected on the LC-MS/MS.

LC-MS/MS was run with positive ion electrospray. The following MRM transitions (Q1/Q3) were used for quantitation of PCPA (199.9/154.1), LX-1032 (575/273.2) and LX1032

metabolite (547.1/272.9). Pharmacokinetic parameters were estimated using the Phoenix software package (Pharsight Corporation, Cary, NC, USA) using non-compartmental analysis and the linear up/ log trapezoidal down calculation method and without weighing. Calculated terms were defined as follows; C_{max}, maximum observed plasma concentration; T_{max}, time of maximum observed plasma concentration; $AUC_{0\text{-}last}$, area under the plasma concentration vs. time curve up to the last measurable concentration, calculated by the log-linear trapezoidal rule.

BON cell assay

The human pancreatic carcinoid cell line BON expressing *tph1* endogenously and secreting 5-HT [3] was obtained from Prof. Dr. Gudrun Ahnert-Hilger (Charité, Berlin). The cells were seeded at a density of 100000 cells/well in a 96-well plate and incubated in complete growth medium overnight at 37°C, 5% CO_2. For the treatment with TPH inhibitors, the full growth medium was replaced with serum free medium. Cells were treated with TPH inhibitors at 12 concentrations for 16 hours. Supernatants were collected and passed through a Multiscreen+HiFlow glass fiber FB plate (Millipore) under vacuum. The samples were dried in a SpeedVac (Concentrator plus, Eppendorf), resuspended in 0.1M acetic acid/ sodium actetate at pH 3.5 (buffer A), separated on a Atlantis T3 column (Waters, #186003726) using high pressure liquid chromatography (Dionex UltiMate 3000 RSLC) running a 2.5 minute gradient from 95% to 10% buffer A at 1.5mL/min (buffer B is acetonitrile). 5-hydroxytryptophan (5-HTP) was quantified with a Dionex UltiMate 3000 FLD-3400RS fluorescence detector λ_{ex} = 280nM and λ_{em} = 330nm. Inhibitory activities of compounds were determined by calculating the IC_{50} value i.e. the concentration of compound needed to inhibit 50% of cellular 5-HTP formation.

Supplementary figures and tables

Supplementary Figure 1: Chromatograms of 5-HT, h-5-HT and h-5-HIAA in rat blood demonstrate method specificity. (Figure **(a)** to Figure **(i)**) show the MRM (/Q3) chromatograms (intensity vs. elution time (min)) generated by LC-MS/MS of 5-HT (178/161) (Figure **(a)** to Figure **(c)**), h-5-HT (171/142.1) (Figure **(d)** to Figure **(f)**) and h-5-HIAA (203/156) (Figure **(g)** and **(h)**) and 5-HIAA (Figure **(i)**). **(a)**, **(d)** and **(g)** are measured from a blood sample from a rat before h-Trp administration. **(b)**, **(e)** and **(h)** are measured from a blood sample from the same rat 1 hour after administration of 30 mg/kg h-Trp. **(c)**, **(f)** and **(i)** are the chromatograms of standards (note no standard of h-5-HIAA was available, however, it co-elutes with the naturally occurring 5-HIAA, see **(i)**).

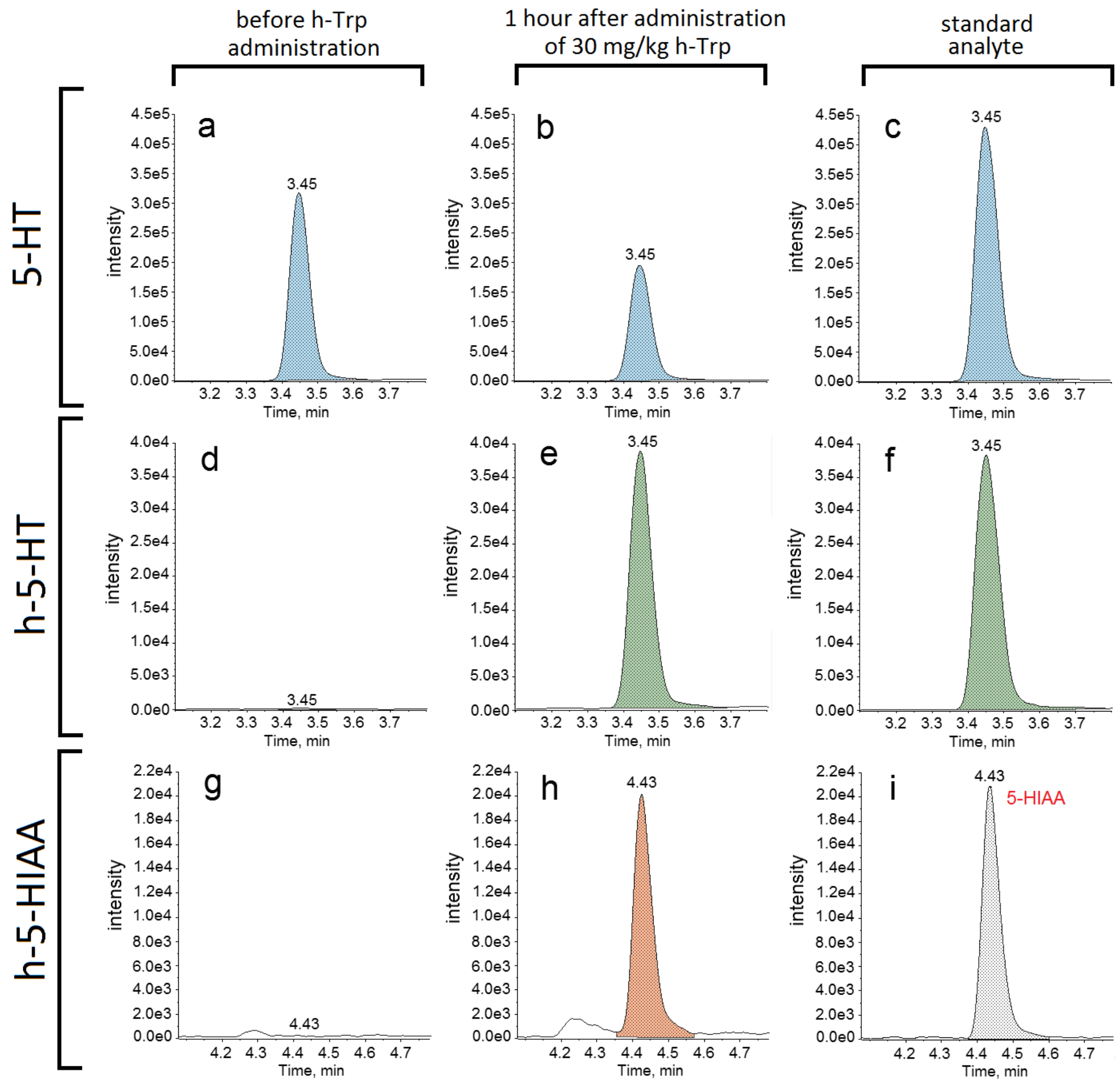

Supplemental Figure 2: Wash-out of stable isotope labeled molecules from rat blood. **(a)** h-Trp and **(b)** h-5-HT. At time 0, rats were given an oral dose of 40 mg/kg h-Trp and serial blood samples were taken. Each trace represents an individual rat (n = 4). For h-5-HT the half-life quoted in the text was generated by fitting the data between 2 and 10 days to a single exponential decay.

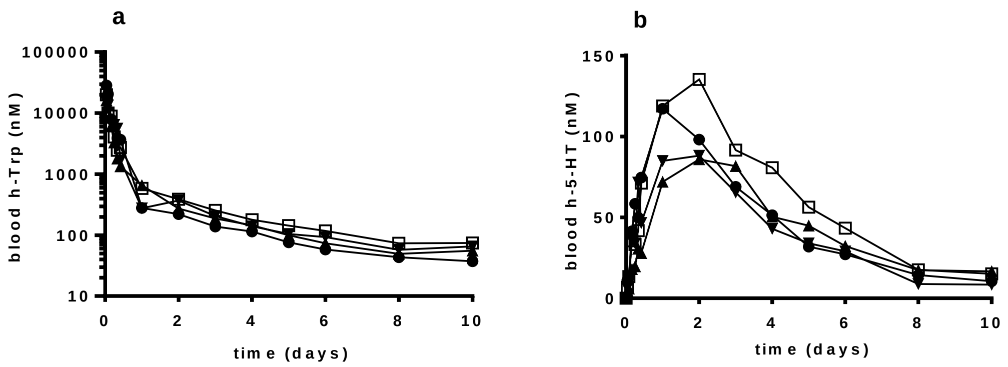

Supplemental Figure 3: Inhibitory effects of a single dose of LX-1032 on conversion of orally administered h-Trp to h-5-HT. **(a)** Blood %-h-Trp; **(b)** blood %h-5-HT; **(c)** blood h-5-HIAA; **(d)** blood Trp; **(e)** blood 5-HT; **(f)** blood 5-HIAA. Solid black circles are vehicle, solid orange circles were pretreated orally with 23 mg/kg LX-1032, 1 hour prior to oral h-Trp administration (n = 4, ± SD).

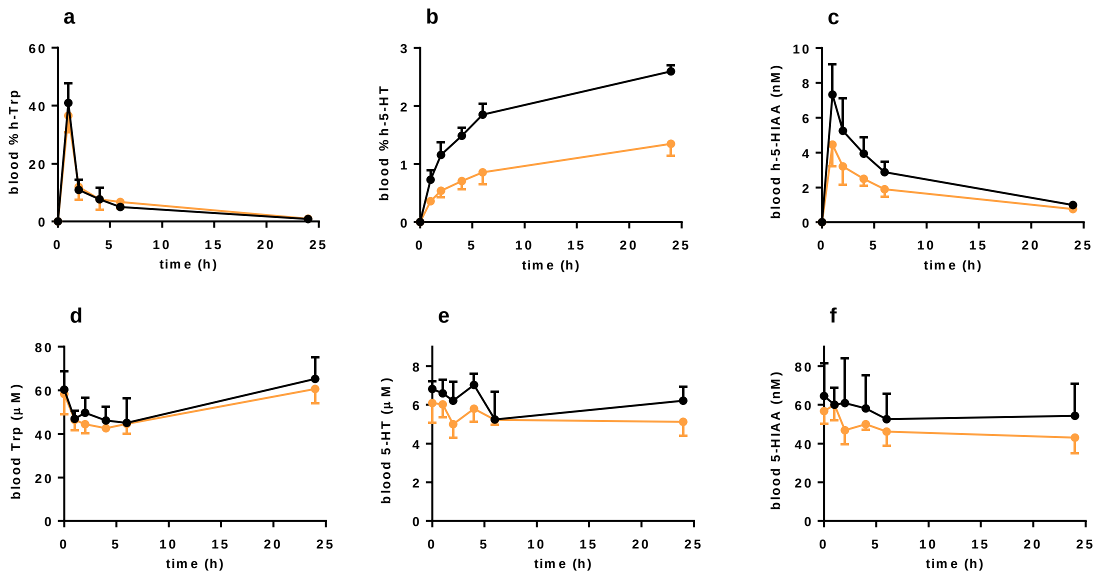

Supplemental Figure 4: Disease induction 7, 14, 21 and 28 days after bleomycin administration in the lung fibrosis model. Measurement at sacrifice of **(a)** Body weight, **(b)** lung weight and **(c)** lung hydroxyproline (HYP) in acid hydrolyzate as a collagen surrogate (n = 4-6 ± SD). A two tailed student's t-test comparing bleomycin to NaCl for individual time points is given; * p < 0.05, ** p < 0.01, *** p < 0.001.

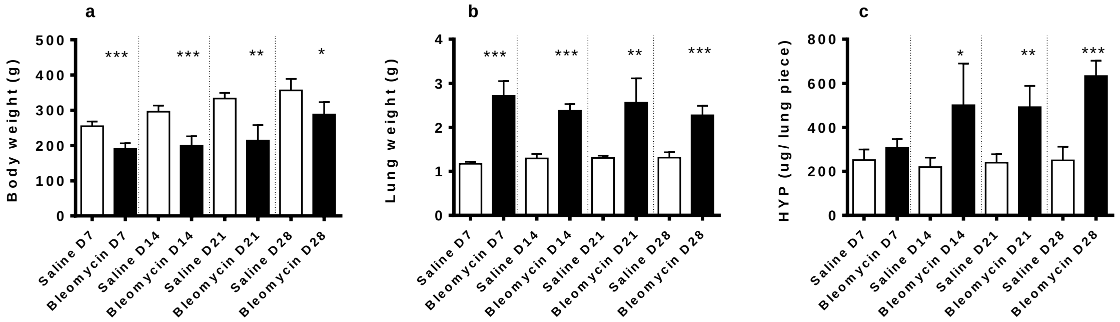

Supplemental Figure 5: Gene expression and metabolite changes of the 5-HT pathway from the lung in the bleomycin lung fibrosis model. **(a)** *Tph1* mRNA; **(b)** *Slc6a4* (*Sert*) mRNA. Additionally, Trp **(c)** and h-Trp **(d)** levels in lung are shown (n = 4-6 ± SD). A two tailed student's t-test comparing bleomycin to NaCl for individual time points is given; * p < 0.05, ** p < 0.01, *** p < 0.001.

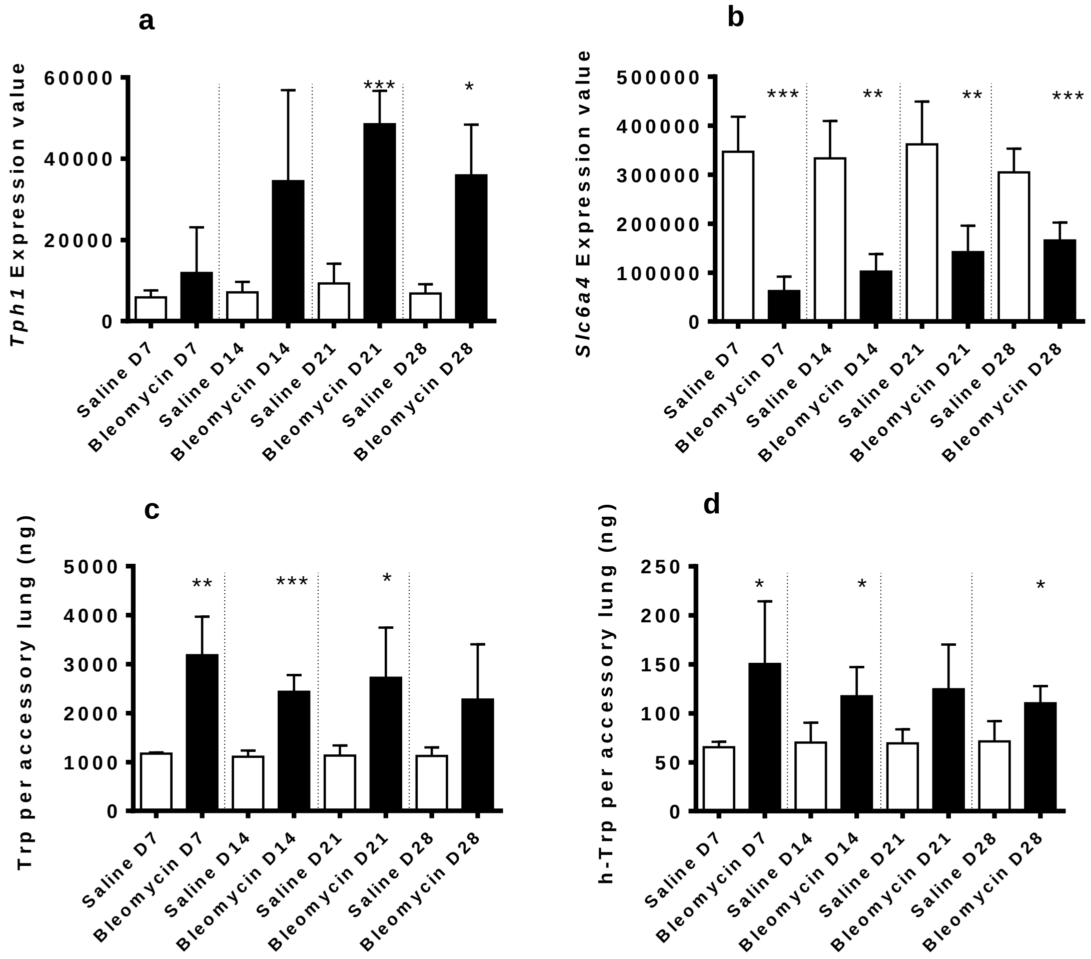

Supplementary Table 1: IC_{50} values and pharmacokinetics of the TPH inhibitors tested for their ability to inhibit production of h-5-HT after the acute administration of h-Trp to rats. Shown are mean IC_{50} values for inhibition of 5-HTP synthesis in BON cells after 16 hours incubation in serum free assay medium. Additionally, pharmacokinetic parameters for the experiments in Figure 2 are given ($AUC_{0\text{-last}}$ and C_{max} as geometric means, t_{max} as median). For 6, 20 and 60 mg/kg of LX-1032, the final timepoint was 10.5 hours after dosing (n = 5-6). For PCPA, the final time point was 25 hours after dosing (n = 4). For LX-1032, values are provided for both the administered ester and the corresponding acid metabolite in square parenthesis.

**Compound**	**Dose**	**BON IC_{50} (nM)**	**$AUC_{0\text{-last}}$ (h*ng/mL)**	**C_{max} (ng/mL)**	**t_{max} (h)**
PCPA	30	1965 (n=2)	315000	25200	3
PCPA	100	1965 (n=2)	1370000	81500	5
LX-1032 [metabolite]	6	12 (n=15) [43 (n=2)]	26 [365]	45 [327]	0.5 [0.5]
LX-1032 [metabolite]	20	12 (n=15) [43 (n=2)]	24 [2380]	27 [842]	1.5 [1.3]
LX-1032 [metabolite]	60	12 (n=15) [43 (n=2)]	78 [2470]	117 [1570]	0.8 [0.8]

Supplementary Table 2: Genes measured in qPCR panel.

Gene	Assay ID
Acta2	Rn01759928_g1
Aqp3	Rn01439528_g1
Bcl2	Rn99999125_m1
C6	Rn00598544_m1
Calm1	Rn00821407_g1
Ccl1	Rn01752376_m1
Ccl2	Rn01456716_g1
Cdh1	Rn00580109_m1
Cma1	rn00565319_m1
Col18a1	Rn01428995_m1
Col1a1	Rn01463848_m1
Col3a1	Rn01437681_m1
Col5a1	Rn00593170_m1
Ctgf	Rn00573960_g1
Cthrc1	Rn00597305_m1
Ctps	Rn00516751_m1
Ctse	Rn01483642_m1
Cxcl2	Rn00586403_m1
Ddc	Rn00561113_m1
Ednrb	Rn00569139_m1
Fhl2	Rn00581565_m1
Fn1	Rn00569575_m1
Gal	Rn01234339_m1
Grem1	Rn01509832_m1
Gsn	Rn01438922_m1
Hbegf	Rn01405658_m1
Hmox1	Rn01536933_m1
Htr1a	Rn00561409_s1
Htr1b	Rn01637747_s1
Htr2a	Rn00568473_m1
Htr2b	Rn00568450_m1
Id2	Rn01495280_m1
Igfbp7	Rn01413246_m1
Il10	Rn00563409_m1
Lcn2	Rn00590612_m1
Lox	Rn00566984_m1
Loxl1	Rn01418038_m1
Loxl2	Rn01466080_m1
Maoa	Rn01430950_m1
Maob	Rn00566203_m1
Mmp12	Rn00588640_m1
Mmp14	Rn00579172_m1

Mmp2	Rn01538170_m1
Mmp7	Rn00689241_m1
Nell1	Rn00675924_m1
Nfe2l2	Rn00477784_m1
Pcsk1	Rn00567266_m1
Pde4a	Rn00565354_m1
Pla2g2a	Rn00580999_m1
Pld2	Rn00583683_m1
Ptger2	Rn00579419_m1
Ptges	Rn00572047_m1
Rac1	Rn01412766_m1
Serpine1	Rn00561717_m1
Slc2a5	Rn00582000_m1
Slc6a4	Rn00564737_m1
Smad6	Rn01766978_m1
Sod1	Rn00566938_m1
Spp1	Rn00681031_m1
Tgfb1	Rn00572010_m1
Tgm2	Rn00571440_m1
Thbs2	Rn02111874_s1
Timp1	Rn01430873_g1
Timp2	Rn0573232_m1
Tnc	Rn01454947_m1
Tph1	Rn01476867_m1
Vcan	Rn01493763_m1
Vegfa	Rn01511601_m1
Wisp1	Rn00581854_m1

control	*28s*	Mm03682676_s1
control	*Actb*	Rn00667869_m1
control	*B2m*	Rn00560865_m1
control	*Gusb*	Rn00566655_m1
control	*Hprt1*	Rn01527840_m1
control	*Pgk1*	Rn00821429_g1
control	*Ppia*	Rn00690933_m1

References

1 Bauer, Y. *et al.* A novel genomic signature with translational significance for human idiopathic pulmonary fibrosis. *Am J Respir Cell Mol Biol* **52**, 217-231 (2015).

2 Vandesompele, J. *et al.* Accurate normalization of real-time quantitative RT-PCR data by geometric averaging of multiple internal control genes. *Genome Biol* **3**, RESEARCH0034 (2002).

3 Tran, V. S. *et al.* Serotonin secretion by human carcinoid BON cells. *Ann N Y Acad Sci* **1014**, 179-188 (2004).
